# *Thirsty? Choose Water!* Behavioural interventions and water stations in secondary schools a two-by-two factorial randomised controlled trial

**DOI:** 10.1186/s12889-018-5685-1

**Published:** 2018-06-26

**Authors:** Nicole Kajons, Michael David, Justine Gowland-Ella, Peter Lewis, Samantha Batchelor

**Affiliations:** 1 Health Promotion Service, Central Coast Local Health District, Level 1, 4-6 Watt Street, Gosford, NSW 2250 Australia; 2 School of Medicine and Public Health, The University of Newcastle, Callaghan, NSW 2308 Australia; 3 Public Health Unit, Central Coast Local Health District, Level 1, 4-6 Watt Street, Gosford, NSW 2250 Australia

**Keywords:** Protocol study, Health promoting schools, School intervention, Obesity, Water consumption, Sugar sweetened beverages, Chilled water stations

## Abstract

**Background:**

Childhood overweight and obesity is a significant public health issue. A key contributing factor is sugar sweetened beverages (SSBs) consumption. Evidence suggests that secondary school students are frequent consumers of SSBs, with high daily consumption. The promotion of water consumption and provision of chilled water stations can reduce SSBs consumption. The *Thirsty Choose Water!* study will evaluate the effectiveness of two interventions, a behavioural intervention, *Thirsty? Choose Water!* behavioural intervention (TCW-BI), that target students through the domains of the health promoting high schools framework, and the second intervention is the installation and promotion of chilled water stations.

**Methods/design:**

This community trial will recruit 60 secondary schools from across three Local Health Districts (LHDs) within New South Wales (NSW). A two-by-two factorial study design will be used to determine the effect of the *Thirsty? Choose Water!* behavioural intervention (TCW-BI), and the installation of chilled water stations. The recruited secondary schools will be randomised and non-blinded to one of four study arms receiving either the TCW-BI or chilled water stations, both interventions, or neither (control group). Baseline measures will be collected including student self-report surveys which will gather data regarding knowledge, attitudes and consumption of water and SSBs, a school profile and an environmental scan. Student surveys will be repeated post the intervention and at follow-up. Regular water meter readings will determine the water flow from the chilled water stations across the study period.

**Discussion:**

There is an increasing body of evidence which suggests that decreasing consumption of SSBs can impact positively on childhood overweight and obesity. However, in the Australian context there are limited studies on how this may occur in the secondary school setting. This study will add to this evidence base and establish the effectiveness of TCW-BI and chilled water stations, either alone or combination on increasing water consumption in adolescents. Information about barriers and facilitators to implementation will be documented. Packages to support the implementation of the TCW-BI as a state-wide initiative will be developed.

**Trial registration:**

Australian and New Zealand Clinical Trials Register ACTRN12618000526279 April 2018.

**Electronic supplementary material:**

The online version of this article (10.1186/s12889-018-5685-1) contains supplementary material, which is available to authorized users.

## Background

### Childhood overweight and obesity

Childhood obesity is a serious public health challenge in Australia, with approximately one in four (27.4%) children aged 5–17 years being overweight or obese [1]. Compared to healthy weight range children, those above a healthy weight are more likely to develop chronic conditions including asthma, Type 2 diabetes, poor emotional wellbeing, poor academic performance [2] and to become obese adults [3].

Compelling evidence suggests sugar sweetened beverages (SSBs) are detrimental to health and contribute to obesity [4]. Recent systematic reviews confirm the link between SSBs consumption and weight gain in both children and adults [5, 6], and that reducing SSBs consumption reduces weight gain in children, particularly in those who are already overweight [7]. During adolescence SSBs consumption increases, with nearly 25% of NSW children aged 12–17 years consuming SSBs at least 5 or more times per week [8]. Substitution of SSBs with water has been associated with a reduction in BMI in early adolescence and increasing the consumption of water has been shown to decrease the incidence of overweight/obesity in children [9]. At a population level, reducing consumption of SSBs is an important modifiable behaviour to address and in combination with other strategies can contribute to alleviating the problem of overweightness and obesity.

There is increasing evidence that addressing this issue in the school setting has merit. A recent systematic review [10] examined 36 interventions in school settings that targeted the individual, the environment or both. This review demonstrated that 70% of these interventions were effective in decreasing SSB consumption, with effectiveness increasing for those interventions that targeted legislation or the environment. Moreover, a systematic review of educational and behavioural interventions to reduce SSB consumption in children and adolescents, found the trend toward reduction in SSB consumption approached statistical significance in those studies conducted within school-based settings (*p* = .06) [11].

Other evidence suggests school based education programs promoting increased water consumption can impact positively on SSBs consumption [12] as can the provision of chilled water stations and water bottles [13] which has been shown to increase water consumption in children and decrease the incidence of overweight in children [13–15].

In view of this evidence, interventions using the health promoting schools framework to address SSB consumption holds promise, and is the basis of this study intervention. A recent Cochrane systematic review demonstrated modest positive intervention effects on range of health indicators for interventions that utilised the Health Promoting Schools Framework [16].

This study is an expansion of an initial pilot project conducted on the NSW Central Coast. In 2016 Central Coast Local Health District piloted a component of the *Thirsty? Choose Water!* behavioural intervention (TCW-BI) targeting the partnership domain of the Health Promoting Schools Framework. Partnering with immunisation nurses, the post-immunisation waiting period was used to deliver messages that promoted water as a preferred drink, and generate discussion regarding SSBs. All year 7 students involved in the secondary school immunisation program were exposed to the intervention. Post-immunisation nurses directed students to participate in “Spouts and Straws” (snakes and ladders), a game which introduces messages about the benefits of water consumption and the negative health effects of SSBs. Preliminary findings suggested students’ knowledge and awareness regarding SSBs increased, with some changes in their water drinking habits. The evaluation indicated high levels of acceptability for students, teachers and immunisation staff and utilising the immunisation program as an opportunity to deliver health messages was considered practical and effective.

This current study expands on this pilot, examining the impact of a TCW-BI addressing all aspects of the health promoting schools framework and/or the installation of chilled water stations on Year 7 secondary school students’ knowledge, attitudes and behaviours regarding the consumption of SSBs and water.

## Methods/design

### Aim

The research aim is to determine if the TCW-BI and/or the provision of chilled water stations increases water consumption in year 7 secondary students and effect changes in students’ knowledge, attitudes and behaviour regarding SSBs.

### Study design

A two-by-two factorial design will be conducted involving secondary schools to determine if the TCW-BI and/or provision of chilled water stations increase water consumption and effect changes in year 7 secondary students’ knowledge, attitudes or consumption of sugar sweetened beverages. Schools recruited to the study will be randomly allocated and non-blinded to one of four study arms. Students and members of the research team will not be blinded to group allocation. A blinded statistician not involved in the trial process will be responsible for final analysis.

### Sample size calculation

Power calculations were conducted to determine the sample size required to detect a change of 5% or more in the primary outcome, that being the proportion of students drinking 5 or more cups of water per day. Change was defined as the difference between the proportion at baseline, assumed to be 40% (as shown in the 2011 School Students Health Behaviour Survey [8]) and at final follow-up. The calculation assumed normality for the outcome, with alpha set at 5% and beta set at 20% [i.e. power at 80%]. In applying power calculations we took into account students being clustered in schools. Using these inputs and prior to an adjustment by design effect for correlated data, it was calculated that an effective sample of 761 students would be needed. Given an assumed intraclass correlation of 0.055 (calculated based on water consumption data from the NSW Schools Physical Activity and Nutrition Survey (SPANS) 2015) and an average school year 7 cohort of 135, this figure was then adjusted by a design factor of 8.37, thus putting the required number of students at 6370, being equivalent to 48 schools. Following adjustments for clustering at the school level (i.e. interclass correlation =0.055) and non-response (i.e. 20%), it was determined that a sample size of 60 schools was required.

### Setting

The study will be conducted in secondary schools (including non-private and private schools) that are within the boundaries of three NSW Local Health Districts (LHDs)(Central Coast, Illawarra Shoalhaven and South West Sydney LHDs). Two of these LHDs are similar in characteristics, which similar population demographics, distance from the hub of Sydney and numbers of secondary schools within them. The third LHD has a significantly higher population, greater diversity and a higher number of schools. To allow the study to progress within these settings ethical approval was obtained through the Hunter New England Human Research Ethics Committee (17/08/16/4.07), the NSW Department of Education (SERAP2017457) and Broken Bay, Sydney and Wollongong Catholic Diocese.

### Sample

All eligible schools within the boundaries of the three participating Local Health Districts will be invited to participate in the study, with the aim of recruiting 60 schools to the trial and randomly allocating 15 schools to each study arm. A sample of 60 schools will be selected from the three LHDs using a proportional stratified sampling technique. All year 7 students at recruited schools will be invited to participate. Schools that already have adequate working chilled water stations in situ (deemed to be one per 300 students) will be excluded from participating in the study. There is limited evidence regarding the ratio of chilled water stations per student, with the figure for this study being informed by guidance from US literature [15, 17, 18] and in consultation with the NSW Department of Education. Schools for special purposes for example schools for students with behavioural difficulties will be excluded from selection.

### Recruitment and study participation

An initial email sent to all eligible schools will inform them of the study. Schools responding to this email expressing interest in the study will be contacted via telephone/email to arrange a follow-up meeting. At this meeting schools will be fully informed of the study protocol, and provided with the study information and consent form. If the school wishes to participate in the study, school level consent will be gained by the principal providing signed consent. Participation of students in the evaluation component of the study will be through an opt-out consent process, where parents must provide written documentation to state they do **not** wish their child to participate in the evaluation (except in Catholic schools within one Catholic Diocese, where an opt-in process will be mandatory).

**Fig. 1:**
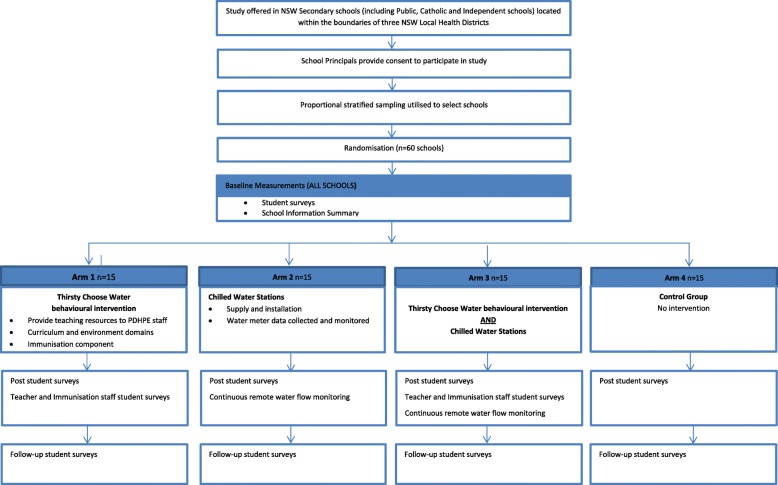
Flowchart of Thirsty Choose Water study

Simple random sampling will be used to select from the pool of consenting schools, the requisite number of schools within each LHD required for inclusion in the study. Once 60 schools are identified for the study sample randomisation of the schools to the study arms will take place. Schools will be randomised by the study statistician using a computerised random number generator. Subsequently, principals will be informed of which study arm they have been allocated to. Those who were not included in the study sample will be informed of this. Following randomisation, baseline measures will be taken, interventions implemented and post and follow-up data collected as per Fig. 1 Flowchart of the study.

### Intervention design

The interventions delivered in this study include a behavioural intervention and the installation and promotion of chilled water stations. Following randomisation, schools will be informed of the study arm to which they have been allocated and the project team will work with the school to progress the allocated intervention.

Schools allocated to the behavioural intervention (Arms 1 and 3) will receive health messages regarding SSBs and the promotion of water as a preferred drink through each domain of the Health Promoting Schools Framework (Additional file 1). Within the curriculum, teaching and learning domain students will receive the intervention through the inclusion of messages in year 7 Personal Development, Health and Physical Activity (PDHPE) lessons and through the TCW website which provides a range of relevant resources. Two PDHPE lesson plans and resources pack have been developed in consultation with PDHPE teachers. These lesson plans outline the key water messages as well as a goal based activity for students to decrease their consumption of SSBs and increase their water consumption. To support the implementation of these lesson plans within the PDHPE curriculum professional development opportunities for teaching staff will also be provided.

The school organisation ethos and environment domain will be addressed by messaging through posters, school newsletters, school social media and other opportunities identified locally by schools to engage students and their families (potentially reaching students in other years). The partnerships and services domain will be addressed through a partnership between schools and local immunisation teams, providing an opportunistic avenue for the TCW messages to be further targeted to year 7 students. Post-immunisation students will participate in the Spouts and Straws game (see Additional file 2), trialled in the project pilot. The TCW - BI intervention will occur from July to December, commencing with the curriculum delivery, and with messages being reinforced trough the school organisation ethos and environment domain and then through the immunisation component.

Schools in Arm 2 and 3 will receive a chilled water station. The installation of chilled water stations in will occur once baseline data has been collected. Local consultation with schools will determine the most appropriate location for the chilled water station, which has access to water, power and drainage. A remote water monitoring system will be added so that the flow of water from these chilled water stations can be recorded. Schools in Arm 3 will receive both interventions. Schools in Arm 4 will be the control group and will receive neither of these interventions, but will complete baseline, post and follow-up measures.

### Outcomes

The primary outcome for the study is self-reported water consumption, with a 5% increase in the proportion of year 7 students drinking 5 or more cups of water per day. This measure is based on data from the 2011 School Students Health Behaviour Survey [8]. The secondary outcome is students’ knowledge, attitudes and consumption of SSBs. See Table 1.

**Table 1:** Primary and Secondary outcomes

Primary Outcome	Secondary Outcome
• Consumption of water	• Students’ knowledge and attitudes regarding water consumption • Students’ knowledge regarding SSBs • Students’ attitudes regarding SSBs consumption • Students’ consumption of SSBs • Students’ purchasing of SSBs • The school environment eg promotion of water messages, reduced access to SSBs

### Data collection

Data collected will include student, water flow and school level data. Student data will be collected from year 7 students at three time points: baseline, post intervention, and at follow-up. Student data will be collected by an online survey monkey or a paper based survey administered in class, dependent on school facilities and resources. Identical surveys will be administered at each time point with additional process evaluation questions at follow-up. A unique identifier (initials and DOB) will be generated in order to match pre- and post-intervention data.

The student survey is composed of items adapted from existing validated survey instruments [19] that will measure students self-reported: Knowledge about consuming water and SSBs; Attitudes towards consuming water and SSBs; Self-reported consumption of water and SSBs; their purchasing of SSBs.

Water flow data will be collected through the installation of water meters and data loggers on chilled water stations to provide an objective measure of water consumed over time. Water meter data will be collected remotely and recorded continuously in real time.

School level data will be collected through a school profile and an environmental scan. School profile data will include information on the type of school (eg Public, Catholic and Independent), size of school (total number and composition of students), the schools Family Occupation and Education Index (FOEI)(a school level index of educational disadvantage related to socioeconomic background) whether the canteen is private or school run and whether the school has vending machines. Students’ access to bottled water and SSBs through environmental scans of the school setting will also occur by identifying within participating schools, what types of drinks are sold at the school (eg canteen and/or vending machines) and what is their closest access point to external sources of SSBs, as measured in walking distance. Given the recent introduction of the NSW Healthy Canteen Strategy, it is expected that the availability of SSBs in the canteen will be increasingly limited.

Data will be also be collected from Arms 1 and 3 regarding the acceptability of the TCW-BI from the perspective of immunisation staff and teachers via a short survey.

#### Statistical analysis plan

Descriptive statistics will be used to summarise study variables. Categorical variables will be reported as counts and proportions. If normally distributed, continuous variables will be presented as means and standard deviations. Otherwise, they will be presented as medians and interquartile ranges.

Baseline characteristics of study arms will be compared to assess similarity at study entry, thereby allowing for the identification of significant imbalances requiring adjustment during analysis. If continuous, differences will be assessed using two-sample t-test or Mann-U Whitney tests for data with non-normal distributions. Categorical variables will be analysed by Pearson chi-squared tests or Fisher’s exact test.

To account for clustering at the LHD and school level and the capture of longitudinal data, multi-level regression models with mixed effects will be developed to estimate interventional effect. For each model building process, variables found to be significantly associated with the outcome measure in the univariable analysis at the 10% level will be retained in the final model. As the primary predictor of interest, intervention effect will be forced into the multivariable model in the form of 4-level categorical variable. Continuous outcome measures will be analysed using linear regression models. For binary outcome measures, analysis will be by binary logistic regression, while multinominal logistic regression will be used to analyse non-binary categorical measures. Diagnostic testing will be conducted on all models to ascertain validity. Each model will be tested for violations of model assumptions, existence of influential points and goodness of fit. Tests for all outcomes will be two-tailed, with levels of statistical significance set at 5%. Statistical analysis will be performed using Stata 15.0 (StataCorp LP, College Station, Texas) statistical software.

### Economic evaluation

The cost consequences of the TCW-BI and/or chilled water station provision to secondary schools across NSW will be determined. To facilitate this, estimates of intervention effect such as water consumption and the purchasing of SSBs will be calculated at baseline and follow-up. Additionally, all direct and indirect cost estimates relating to the establishment and continuation of the interventions will be collected and assessed from a healthcare perspective. Decision tree modelling, populated by these effect and cost estimates will then be used to estimate the intervention impact in the form of incremental cost-effectiveness ratios (ICERs), accompanied by their respective 95% confidence intervals. Discounting will not be incorporated into the modelling due to follow-up being less than 1 year. One-way sensitivity analyses will be conducted by varying model inputs within a range representing high and low plausible values. Monte Carlo simulation will be used to assess the robustness of our results by varying all model inputs simultaneously over 10,000 iterations in Ersatz (www.epigear.com).

## Discussion

Addressing overweight and obesity in adolescents is a significant public health challenge. Whilst a myriad of factors are implicated in addressing this complex issue, the reduction of SSBs consumption and the promotion of water consumption within this population is one emerging approach to address this issue, with school based interventions being increasingly utilised. In the Australian context, however, there is little evidence of this. Of the 36 interventions identified in a recent systematic review of school-base interventions only two were identified as Australian studies and these both focussed more broadly on healthy eating and physical activity interventions, rather than more specifically on SSB consumption [10]. In this context, this current study adds to the evidence, generating new knowledge of how to increase water consumption and decrease SSB consumption amongst adolescents, in an Australian school setting.

The primary aim of the study is to test the effectiveness of number of interventions to increase year 7 secondary student water consumption. These interventions include the delivery of a behavioural intervention delivered within the Health Promoting Schools Framework, the installation of chilled water stations or both. Within the current literature many interventions have a behavioural approach or address the environment, but few studies incorporate both [10], and in reviewing the evidence the provision of both these interventions within one study has not been tested previously in the Australian context or with this age group, and this is a strength of this study.

In addition to filling this evidence gap, there are several strengths to this study. The study design is novel in that it utilises a two by two randomised factorial design, in a real world school setting. To date randomised controlled trials have not been the predominant approach to test interventions regarding SSBs in the school setting. The recent systematic review by Vezina-Im et al. [10] reported only 36% of studies utilised this design. The inclusion use of a factorial design is also beneficial and increases the efficiency of the study by simultaneously investigating two interventions by including all schools in both analyses and to consider both the separate effects of each intervention and the benefits of receiving both interventions together [20]. A further strength of the study design is the testing of the intervention across differing health regions as well as across different secondary settings (including public, private and catholic schools). It is also anticipated that the findings from the study will be robust and generalisable to other school populations, both nationally and internationally, as the inclusion of remote water meter monitoring on chilled water stations will provide an objective, continuous measure of water consumption and will assist to validate the findings from the student self-report survey regarding their water consumption.

This study provides an opportunity to build on a novel approach of using a pre-existing health intervention (the immunisation period) to deliver a choose water message. The provision of both a behavioural component and environmental changes (eg installation of chilled water stations) within the study appears to align well with current evidence that shows the inclusion of both of these approaches produces the most efficacious outcomes in decreasing SSB consumption. It is anticipated that the findings from this study will not only inform NSW Health and the NSW Department of Education of successful scalable interventions, but also contribute to the broader evidence base regarding the health impacts of these interventions in improving water consumption and influencing overweight and obesity in secondary school students.

## Additional files

Additional file 1: Thirsty Choose Water and the Health Promoting Schools Framework. (PDF 207 kb)

Additional file 2: Spouts and Straws game. (PDF 320 kb)

## Abbreviations

DOB: Date of birth
FOEI: Family occupation and education index
ICERs: Incremental cost-effectiveness ratios
LHDs: Local Health Districts
NSW: New South Wales
PDHPE: Personal development, health and physical activity
SERAP: State education research applications process
SPANS: Schools physical activity and nutrition survey
SSB: Sugar sweetened beverage
SSBs: Sugar sweetened beverages
TCW-BI: *Thirsty? Choose Water!* Behavioural Intervention
US: United States of America
