# Supplementary figures and images for: Thirsty? Choose Water! Behavioural interventions and water stations in secondary schools a two-by-two factorial randomised controlled trial
Source: BMC Public Health. 2018 Jun 26;18:788. doi: 10.1186/s12889-018-5685-1 (PMC6019217; doi:10.1186/s12889-018-5685-1)

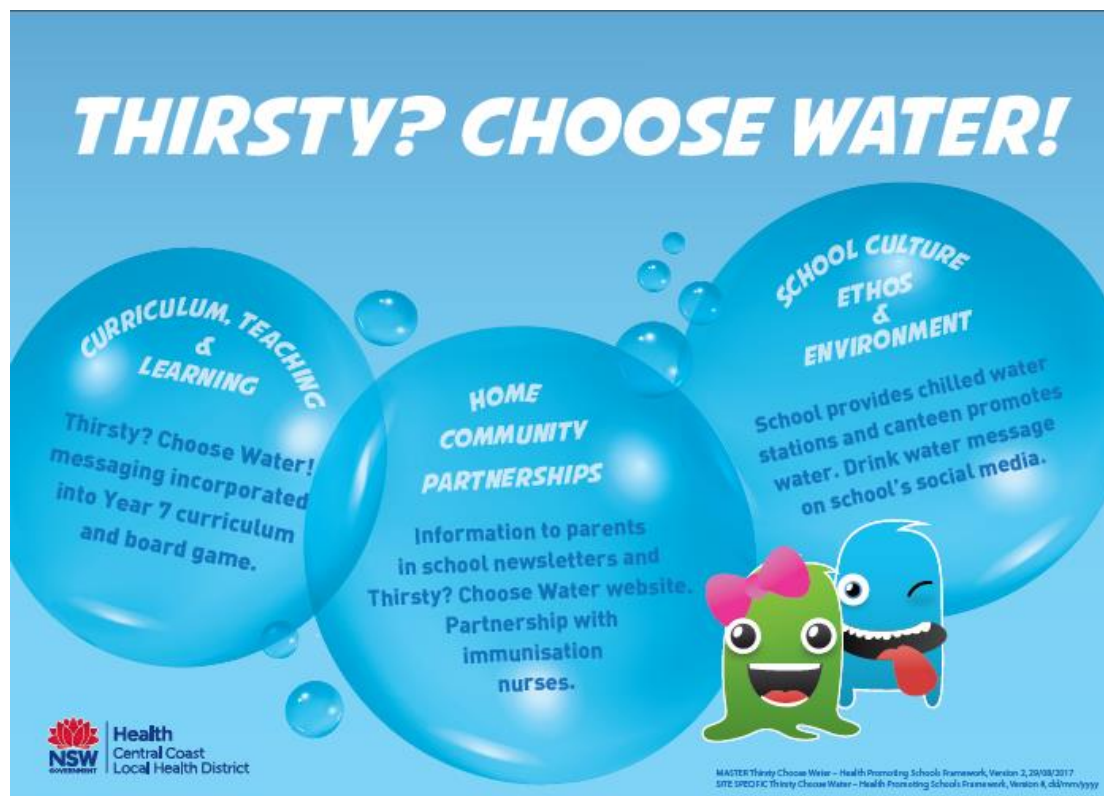

Supplement: Supplementary file 1 — Thirsty Choose Water and the Health Promoting Schools Framework. (PDF 207 kb) [file 12889_2018_5685_MOESM1_ESM.pdf]
